# Hydrological Connectivity and Local Environment Alternately Drive Spatial Structure of Floodplain Aquatic Community Across Seasons

**DOI:** 10.1002/ece3.70880

**Published:** 2025-02-24

**Authors:** Hiromi Uno, Shunsuke Utsumi, Kentaro Morita, Osamu Kishida, Md. Khorshed Alam, Junjiro Negishi

**Affiliations:** ^1^ Faculty of Environmental Earth Science Hokkaido University Sapporo Hokkaido Japan; ^2^ Graduate School of Life Sciences Tohoku University Sendai Japan; ^3^ Field Science Center for Northern Biosphere Hokkaido University Sapporo Hokkaido Japan; ^4^ Uryu Experimental Forest, Field Science Center for Northern Biosphere Hokkaido University Horokanai Hokkaido Japan; ^5^ Atmosphere and Ocean Research Institute The University of Tokyo Kashiwa Chiba Japan; ^6^ Tomakomai Experimental Forest, Field Science Center for Northern Biosphere Hokkaido University Tomakomai Hokkaido Japan; ^7^ Wakayama Experimental Forest, Field Science Center for Northern Biosphere Hokkaido University Wakayama Japan

**Keywords:** amphibian, assemblage, benthic macroinvertebrate, fish, floodplain, plankton, season, β‐Diversity

## Abstract

Seasonal changes in the environment often strongly influence biological communities. In environmental transition zones, or ecotones, the environment fluctuates over time between two different types of environments and the seasonal change is more pronounced. Although emphasis has been placed on the spatial variation of biota along environmental gradients, seasonal change has not been well studied despite the seasonal nature of many ecotones. In this study, we investigated seasonal shifts in aquatic community structures of floodplain waterbodies characterised as transitions between lotic and lentic environments, and further investigated the biological processes behind the shift. We observed a clear seasonal shift of community structure in floodplain waterbodies. From the spring snowmelt season to the summer low flow season, the aquatic community structure was largely driven by the hydrological connectivity to the river, represented as the timing of the lotic–lentic transition during the seasonal flood recession. In contrast, after a few months of summer low flow period, the effect weakened over time, and the communities were structured based more on the basis of the local environment. The seasonal shift was largely explained by the change in amphibian and aquatic insect larvae, the main members of the floodplain aquatic assemblage, which metamorphose and emerge from the water during the summer period and then redistribute in different ways more strongly influenced by local environmental factors such as water body size, temperature and dissolved oxygen levels. Given that biota in ecotones occupy the habitat for a limited time due to the severe environmental fluctuations, such seasonal changes as we observed in this study may be widespread in ecotones. Landscape and local environmental conditions could alternately shape community structures in different seasons. Further attention to the temporal aspects of community structure is needed for community studies and for conservation.

## Graphical abstract

Our study shows that the landscape‐scale environmental gradient represented as ecotone and as local environmental factors can alternately determine the spatial structures of the floodplain biota across seasons. The study highlights the understudied yet important linkages of spatial and temporal biotic assemblages and advance understanding of ecology.

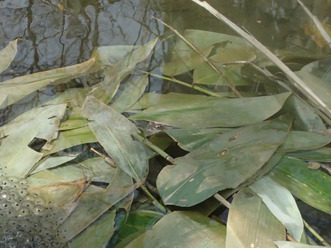

## 1 Introduction

All organisms in nature are challenged to live in spatially and temporally variable environments at various scales (Levin 1992). Local environments as well as the landscape positions and histories drive local communities through environmental filtering and dispersal limitation from a regional species pool (Gilbert and Lechowicz 2004; Thompson and Townsend 2006). Biological communities can vary both in space and time, and knowing what is driving the spatial and temporal structures of communities in natural landscapes is essential to understanding regional community structures (Leibold et al. 2004; Gounand et al. 2018).

**FIGURE 1:**
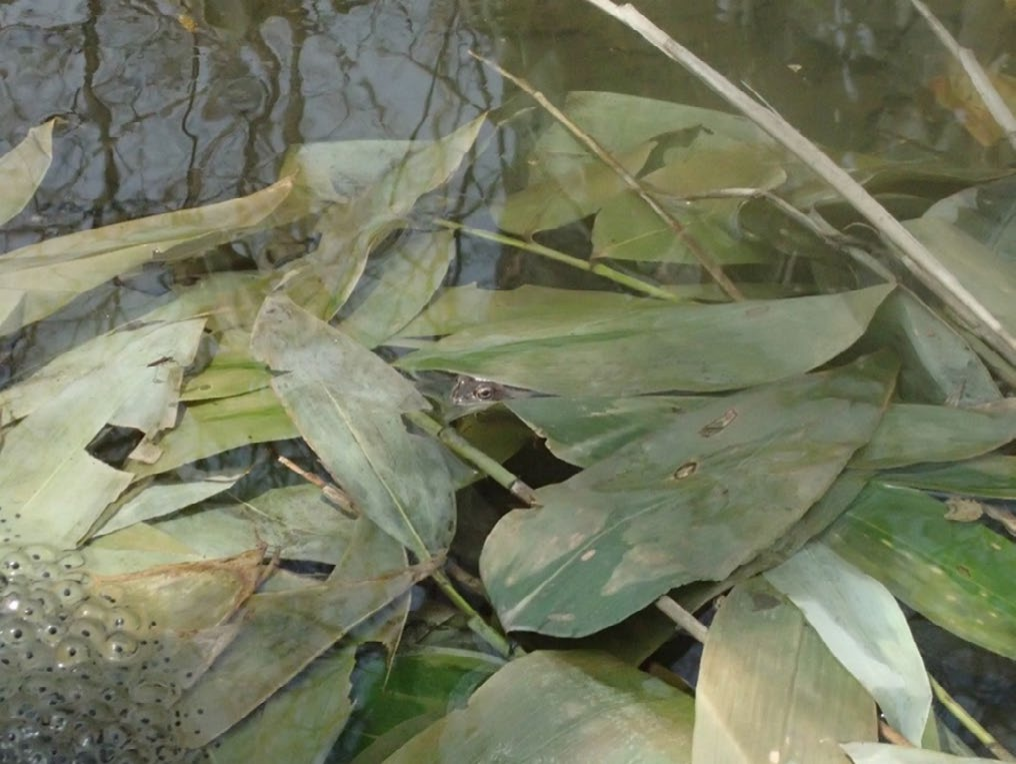
One of the most representative seasonal inhabitant of the studied floodplain waterbodies, *Rana pirica*. Adults of *R. pirica* live in land in most of the seasons, and come to the floodplain and lay their eggs in preferable waterbodies during snowmelt flood recession. Larval *R. pirica* grow in the waterbodies their parents select.

**FIGURE 2:**
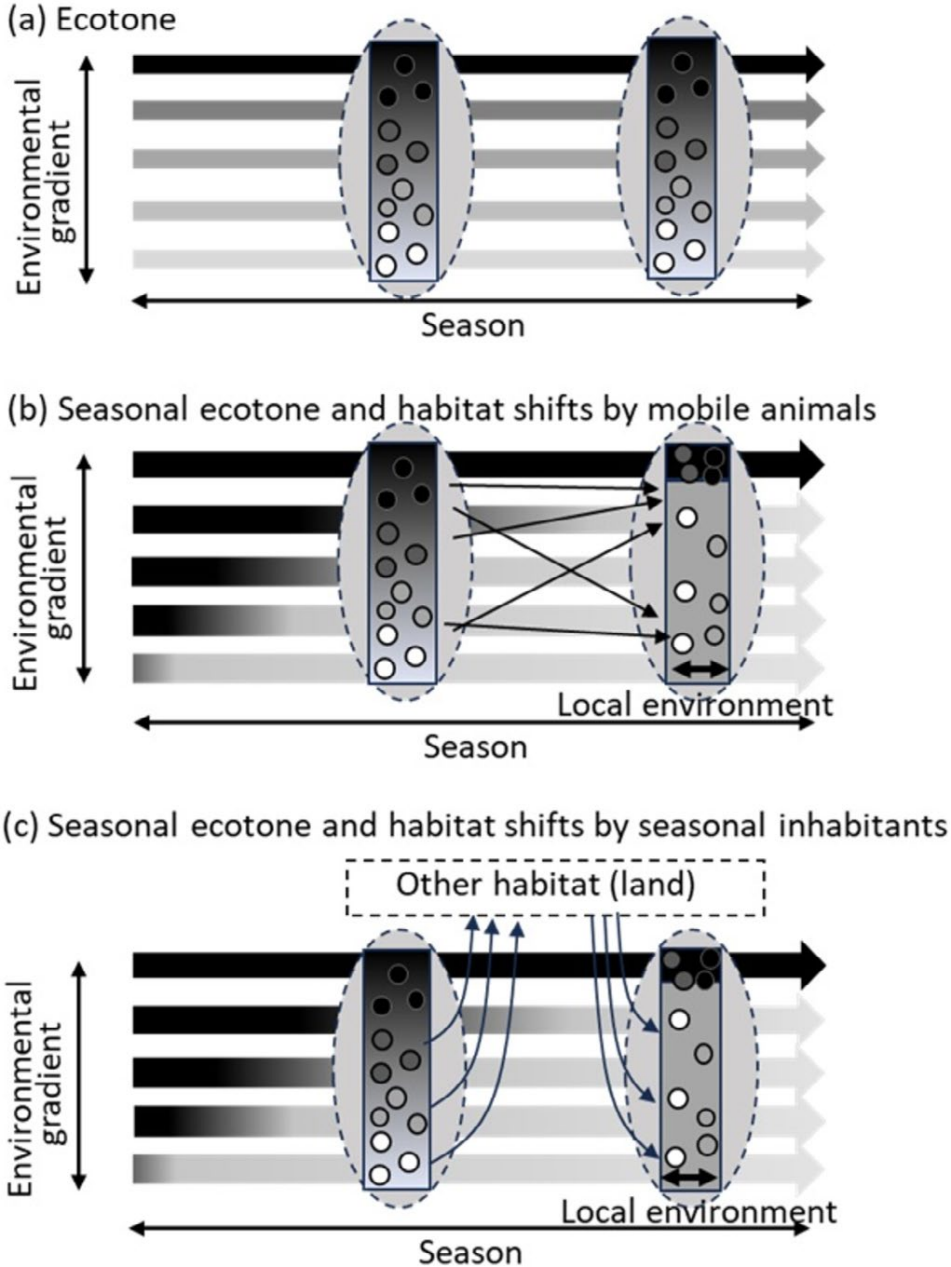
Conceptual diagrams of (a) ‘ecotone in traditional view’ and (b, c) ‘seasonal ecotone and associated seasonal shift in community structure’ proposed in this study. Colours of arrows in the back indicate environment, black and white as two extremes (cf. terrestrial and aquatic, lotic and lentic). In the ecotone in traditional view, there is environmental gradient from one extreme to another at all times, whereas seasonal ecotone is represented more as habitats that experience two different environments seasonally at different proportions of time. Communities in the seasonal ecotone would exhibit seasonal shifts as the environment change, (b) by movements between habitats or (c) by seasonal occupations by different organisms.

Ecotones at the margins of two different environments provide unique and precious habitats for various organisms and provide important ecological functions (Figure 1, Risser 1995; Smith et al. 1997). They are characterized as spatial environmental gradients, but they are often also temporally dynamic. Integral understanding of the spatial and temporal dynamics should largely help understanding ecosystems at ecotones. In fact, in many ecotones, the environment commonly shifts between two different types temporally, and the environmental gradient along the transition zone is represented by the proportion of time that each type of environment is present (seasonal ecotone) rather than a static environmental gradient in space (traditional ecotone) (Figure 2). For example, the environmental gradient of the aquatic–terrestrial ecotone may be formed by the duration of submergence (van der 1990). Many studies have shown species turnover of the biological communities in the ecotone along with the environmental gradient from one extreme to another at a point in time (Tockner, Malard, and Ward 2000; Peterson and Reich 2008). However, when taking into account the temporal nature of the environmental gradient in the ecotone, which is primarily driven by variation in the timing or frequency of environmental transitions, the magnitude of the effects of landscape positions on the local communities should vary with time elapsed since the environmental transition. The large seasonality in abiotic and biotic factors in the ecotone would cause spatial structures of communities to shift seasonally (Figure 2).

Natural floodplains are one of the most representative seasonal ecotones, as transitions between aquatic and terrestrial ecosystems (Junk, Bayley, and Sparks 1989), and transitions between lotic and lentic ecosystems (Tockner, Malard, and Ward 2000). On a natural floodplain, there are many extant and paleo‐side channels as a result of channel migration and abandonment. These waterbodies represent typical lotic and lentic transitions, as the river water flows through such waterbodies above a threshold river discharge. They have diverse degrees of hydrological connectivity to the river main channel, and the duration or frequency of river water flowing through the waterbodies varies (Amoros and Roux 1988; Tockner, Malard, and Ward 2000). In many temperate floodplains, spring snowmelt causes long‐lasting floods, and many biota take advantage of the temporal changes. Although water flows through some waterbodies for prolonged periods during the flood season, the period of flow is limited or absent for other waterbodies. Such gradients in connectivity support spatially variable biological assemblages from lotic to lentic extremes with gradual species turnover after flooding (Uno et al. 2022). Although there is a general prediction that the aquatic communities in such waterbodies vary along with the hydrological connectivity gradient (Van den Brink, Katwijk, and Van der Velde 1994; Morand and Joly 1995; Tockner, Malard, and Ward 2000; Gallardo et al. 2014), seasonal components and formation processes of spatial structures in the aquatic communities in a floodplain have not been well studied.

Diverse aquatic biota inhabiting floodplains exhibit various behaviours and life cycles. Although the spatially heterogeneous and temporally dynamic floodplain environment challenges some biota and some habitat may function as ecological traps, some biota are adapted to such dynamic environments and take advantage of them. Short‐lived animals such as plankton may quantitatively respond to the environment locally (Baranyi et al. 2002). Mobile animals such as fishes may move between waterbodies as the environment changes (Figure 2b, Armstrong et al. 2013). Amphibious animals such as many aquatic insects and amphibians may only seasonally inhabit the floodplain waterbodies, and their distribution may correspond to the environment of the specific season (Figure 2c, Hamer et al. 2023).

In this study, we investigate how biological communities persist in seasonal ecotones across seasons, as represented by floodplain aquatic communities. We test a hypothesis that the primary driver of biological communities in a seasonal ecotone changes from ‘environmental gradient’ to ‘local environment’ with time after the environmental transition (Figure 2). In the case of floodplain aquatic water bodies, we predicted that the primary driver of aquatic communities changes from ‘seasonal hydrological connectivity’ to ‘physical/chemical properties of each waterbody’ with time after the flood recession. To test these hypotheses, we studied how four biological groups with different life histories, including plankton, benthos, fishes, and amphibians, shifted their habitats in a floodplain with time after snowmelt flood recession. We aimed to synthesize seasonal responses of various animals in seasonal ecotones and identify important environmental characters and habitat connectivity for biological communities in ecotones.

## 2 Methods

### 2.1 Study System

**FIGURE 3:**
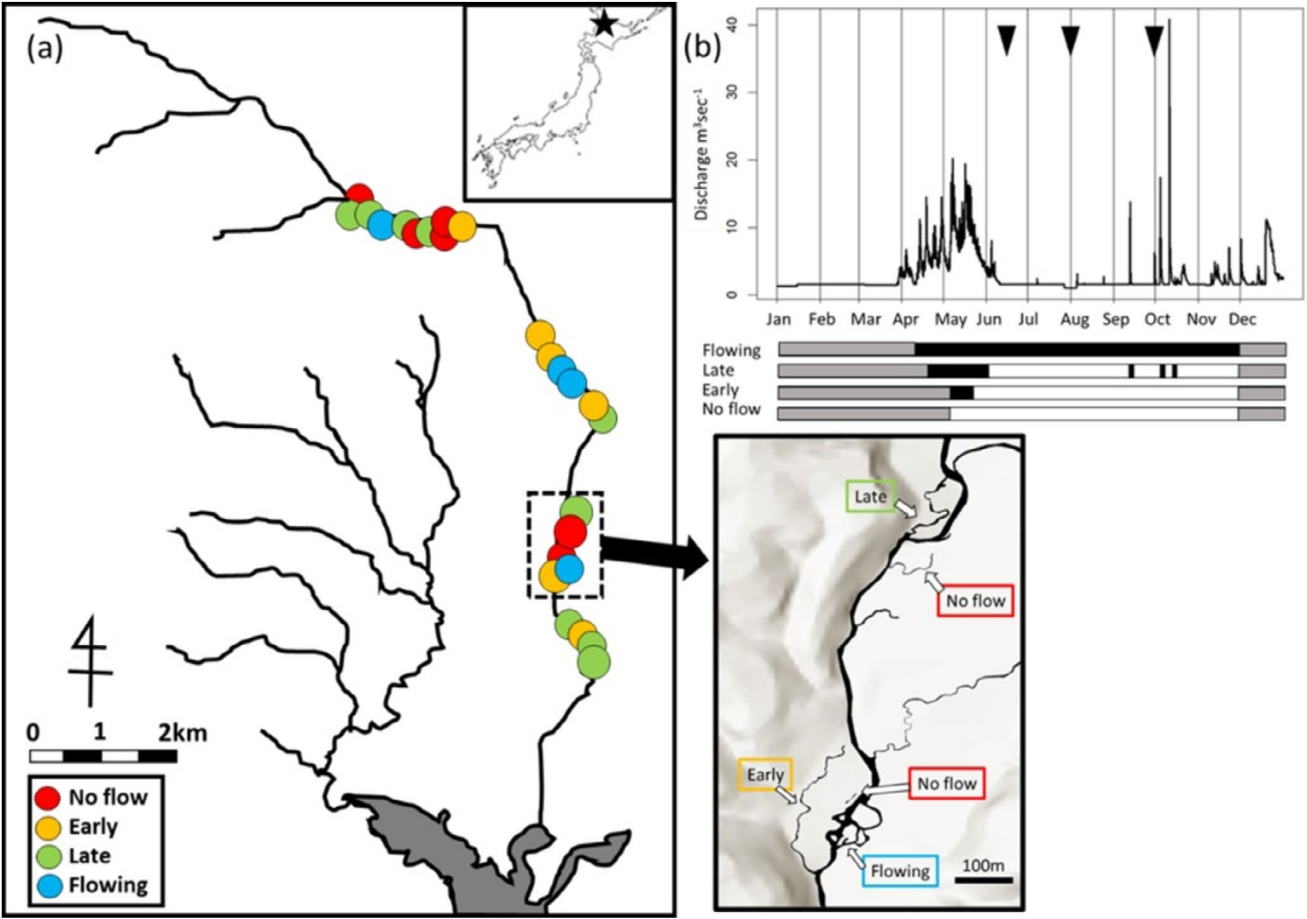
(a) Locations of study sites along the Butokamabetsu River, Hokkaido, Japan. Sampling was conducted at 25 paleo‐ and extant‐side channels of the Butokamabetsu River. Sampling sites were categorised in four hydrological connectivity groups based on the presence/absence of flow at flood peak, flood recession timing and presence of flow at low flow (represented by colours). Some of the representative sites are shown in larger view to show general planform of the Butokamabetsu River. (b) Seasonal change in the discharge of the Butokamabetsu River from April to December 2021 (Data provided by Hokkaido Electric Power Company). The three triangles indicate the timings of spring, summer and autumn surveys. The bands at the bottom show the hydrological connectivity of each habitat category across seasons: grey indicates ‘ice covered’, black indicates ‘flowing’ and white indicates ‘no flow’.

The study was conducted on the Butokamabetsu River floodplain, located in the Hokkaido University Uryu Experimental Forest, northern Japan (44°24′N, 142°13′E) (Figure 3, described in detail in Uno et al. 2022). The mainstem of the Butokamabetsu River, which is about 10 m wide under summertime low‐flow conditions, has many extant‐ and paleo‐side channels. Extant‐side channels remain connected to the mainstem even during periods of low flow and the water remains flowing year‐round. Paleo‐side channels are old side channels that have become isolated from the mainstem through the loss of their upstream connection with the river because of fluvial sediment and wood accumulation and flood dynamics. Many paleo‐side channels, including our study sites, harbour stagnant water during low flow (also called oxbow lakes). Floodwater flows into some of the paleo‐side channels at high flow, and the water keeps flowing through these channels until the mainstem water level drops to a level specific to each paleo‐side channel, depending on the side channel's morphology. Therefore, a gradient of hydrological connectivity to the river exists among waterbodies on the floodplain: extant‐side channels, seasonally connected paleo‐side channels and permanently isolated paleo‐side channels.

Snowmelt typically occurs from April to May, and the river discharge is elevated over this period (Figure 3). Various aquatic animals, including amphibians, fishes, aquatic insects and plankton, take advantage of this yearly snowmelt flooding for dispersal, spawning opportunity and other functions and Uno et al. (2022), have previously documented that the hydrological dynamics over the snowmelt period shape the spatially heterogeneous aquatic communities in the floodplain. Besides snowmelt flooding, the area also experiences summer spates due to rain, which can cause higher peak river discharge but are much shorter in duration than the snowmelt flood and usually last only for a few days.

### 2.2 Survey

To examine how floodplain aquatic communities change with time after the flood recession, we conducted three surveys after the snowmelt flood: spring (14–18 June 2021), summer (25–29 July 2021) and autumn (27 September–1 October 2021). A total of 25 waterbodies (including both paleo‐ and extant‐side channels) with different degrees of connectivity to the mainstem in five spatial blocks along a 10‐km segment of the Butokamabetsu River were selected (Figure 3). These are the same set of sites described in Uno et al. (2022).

To evaluate the hydrological connectivity of each study site to the river mainstem, we set a time‐lapse camera at the upstream end of each studied paleo‐ or extant‐side channel prior to the snowmelt flood season (early May) and monitored whether each waterbody was flooded by the river water, and if so, for how many days. We have previously confirmed that once mainstem river water flowed in from the upstream end, the water flowed through the waterbodies and out from the downstream end. Some waterbodies experienced back flooding, whereby the water entered from the downstream end, but we defined the environmental gradient based on the upstream connectivity, not the downstream connection, because only the upstream connection initiated the flow of floodplain waterbodies. On the basis on these observations, we categorised the study sites into four connectivity categories (Figure 3). The four categories are ranked categories, and the connectivity is ‘Flowing’ > ‘Late’ > ‘Early’ > ‘No flow’, based on the number of days the water flow through each waterbody (Figure 3b). ‘No‐flow’ sites were never flushed by floodwaters because the upstream end of the paleo‐side channel was always closed. At the ‘Early’ and ‘Late’ sites, each waterbody was flushed by flow at peak discharge and isolated from the mainstem at low flow but differed by the timing of flow cessation. They were differentiated by the timing of the flow cessation, characterised as a seasonal ecotone, before 30 May 2021 at ‘Early’ sites and after 1 June 2021 at ‘Late’ sites. At ‘Flowing’ sites, flow was continuous throughout the study period.

The survey targeted four faunal groups: plankton, benthos, nekton (fishes) and amphibians. Benthic invertebrates were sampled with Surber net samplers (25 × 25 cm). Three samples (total 0.1875 m^2^) were collected at each site on each sampling date and combined. Benthic samples were immediately sieved through a 0.5‐mm mesh and preserved in 99% ethanol for later sorting. Fishes were captured with a backpack electrofishing unit (Model 12B; Smith‐Root Inc., Vancouver, Washington, USA) using a pulsed direct current setting (300–400 VDC). A crew of two or three study participants sampled in an upstream direction. The entire area of small waterbodies or the first 20–120 m of paleo‐side channels at the site longer than 120 m was sampled by the two‐pass method. All fishes collected in the surveys were identified to species and released back to the same site alive. The fish catch per unit effort was calculated by dividing the fish count by the total habitat area sampled. The density of amphibian larvae was binomial (very high or zero) because their presence depended on whether their adults laid egg masses, which contain many eggs. Therefore, for amphibians, we recorded the presence/absence of their larvae rather than their density by visual investigation during the electrofishing survey. For plankton, 10 L of water was collected by scooping water from all around the respective waterbody, filtered through a 70‐μm‐mesh plankton net and preserved in 2% glutaraldehyde for zooplankton analysis.

As the local environment, we measured pH and conductivity with a portable pH/COND meter (D‐74; HORIBA, Kyoto, Japan) and dissolved oxygen and water temperature with a portable multimeter (HQ‐30d; HACH, Loveland, Colorado, USA) during each sampling event (Table 1). Furthermore, to characterise the physical environment, each time we estimated the pond area, average depth and flow in ponds, and recorded the presence of surface water connection to the river mainstem at the downstream end of the waterbody. Additionally, during the autumn survey, we recorded the presence/absence of external surface water input from the mountain side, mean grain size of the sediment and depth of the organic layer at each site.

**TABLE 1:** Summary of local environmental variables for four connectivity categories.

	No flow		Early		Late		Flowing	
	Mean	SE	Mean	SE	Mean	SE	Mean	SE
Number of sites	6		6		9		4	
Flow at flood peak	N		Y		Y		Y	
Flow on 1 June	N		N		Y		Y	
Flow on survey dates	N		N		N		Y	
Grain size (mm)	21	16	37	20	47	17	53	28
Spring								
Area (m^2^)	69.2	25.0	95.9	25.3	112.5	26.1	188.7	59.5
Depth (m)	0.26	0.08	0.25	0.07	0.24	0.04	0.16	0.05
Downstream end connection	2/6		3/6		7/9		4/4	
Temperature	14.4	1.1	16.7	1.0	14.1	1.1	15.3	0.9
Dissolved oxygen (mg L^−1^)	5.1	1.1	7.4	1.2	5.3	1.1	10.5	0.2
pH	6.4	0.1	6.7	0.1	6.8	0.2	7.4	0.1
Conductivity	7.7	1.3	7.4	0.6	7.1	0.2	5.7	0.1
Summer								
Area (m^2^)	43.0	23.2	63.9	18.5	75.3	22.4	110.6	33.9
Depth (m)	0.15	0.06	0.23	0.06	0.21	0.04	0.14	0.03
Downstream end connection	1/6		2/6		4/9		3/4	
Temperature	21.2	0.8	21.9	0.7	19.6	0.9	20.3	1.2
Dissolved oxygen (mg L^−1^)	2.6	0.8	4.4	1.1	3.6	0.8	8.0	1.5
pH	6.8	0.1	6.9	0.1	7.0	0.1	7.4	0.1
Conductivity	7.8	1.3	10.0	2.9	8.2	0.3	7.8	0.8
Autumn								
Area (m^2^)	51.5	23.8	97.7	23.3	98.6	28.0	130.3	37.7
Depth (m)	0.20	0.04	0.24	0.03	0.25	0.04	0.16	0.04
Downstream end connection	2/6		2/6		2/9		4/4	
Temperature	11.9	0.8	11.9	0.7	11.6	0.5	12.2	0.5
Dissolved oxygen (mg L^−1^)	5.1	2.1	5.3	1.3	4.5	1.1	10.4	0.4
pH	7.0	0.3	7.4	0.2	7.0	0.2	7.5	0.3
Conductivity	13.9	3.8	9.8	1.8	12.8	1.9	8.1	1.6

### 2.3 Data Analysis

We used multivariate techniques to examine the influence of connectivity and seasons on amphibians, fishes, benthos and plankton assemblages, and also all biota together. Density data of all faunal groups were standardized using the decostand (XX, method = ‘max’) function in the vegan package (Oksanen et al. 2013) in R prior to analysis so that the density data of each taxon range between 0 and 1. We then analysed the assemblage data at 25 sites in three seasons (as 75 data) by non‐metric multidimensional scaling (NMDS) using the Bray–Curtis dissimilarity index as pairwise β‐diversity values. We applied two‐way PERMANOVA procedures to the β‐diversity values to test for statistical differences in the biota by the connectivity gradient and by seasons using the adonis function in the vegan package (Oksanen et al. 2013). When there were significant interactions of connectivity and seasons, we applied PERMANOVA for the 25 data of each season to examine the effect of connectivity on the assemblages in each season.

We then used generalised dissimilarity modelling (GDM) to analyse the relative contribution of connectivity and local environment to the β‐diversity in each season. GDM is a non‐linear extension of matrix regression for analysing and predicting patterns of assemblage dissimilarity in relation to environmental gradients. Dissimilarities between all possible pairs of assemblages were calculated and were modelled as a function of environmental dissimilarities, assuming their non‐linear relationships (Ferrier et al. 2002, 2007). We applied GDM to 25 assemblage data sets for each season. Using the gdm package (Manion et al. 2017), we first converted each dissimilarity matrix and the environmental data to site‐pair format, applied GDM with the function gdm and estimated the importance of environmental data using the function gdm.varImp. As connectivity variables, we used the four connectivity categories ‘No flow’, ‘Early’, ‘Late’ and ‘Flowing’ based on the presence of inflow from the main stream over time, as described above. As environmental variables, we included the area and depth of each pond, average flow velocity, downstream connectivity of each pond to the river, grain size, water temperature and dissolved oxygen. These environmental variables, including water temperature and dissolved oxygen, were measured only at the time of sampling at most sites, but we confirmed that the diurnal variation was smaller than the variation between sites by repeated measures at some sites. pH and conductivity were not included in the analysis because of their high association with DO (positive) and temperature (negative).

To partition variation in biotic assemblages, each group of explanatory variables was first screened with forward selection with a Monte Carlo randomisation test, and only variables significantly related to assemblage structure in forward selection were retained in the final models. We then carried out three model fittings for each assemblage data set along with a different set of environmental data matrices. Matrix‐(I) was constrained by both connectivity and local environments (*a* + *b* + *c*), where *a* describes pure effects of environmental variables, *b* shared environmental and connectivity effects and *c* pure effects of connectivity; matrix‐(II) was constrained by environmental variables only (*a* + *b*); and matrix‐(III) was constrained by connectivity only (*b* + *c*). Variation in assemblage structure was subsequently partitioned into shared environment and connectivity (*b* = [*a* + *b*] + [*b* + *c*] – [*a* + *b* + *c*]), pure local environment (*a* = [*a* + *b*] – [*b*]), pure connectivity (*c* = [*b* + *c*] – [*b*]) and unexplained fractions (*d* = 1 – [*a* + *b* + *c*]) (Borcard, Legendre, and Drapeau 1992; Legendre and Legendre 1998).

The result of variation partitioning shows that local environments have a strong influence on the aquatic community structures in autumn. To better understand the processes how local environments structure the aquatic community structures in autumn, we carried out a canonical correspondence analysis (CCA) additionally. As the processes that community structures shift, we considered new recruitment of amphidromous organisms such as aquatic insects and amphibians (Figure 2c) because most floodplain waterbodies were isolated from each other during summer low flow and movements between them (Figure 2b) were obviously very limited. Therefore, we carried out CCA with autumn benthic macroinvertebrate and amphibian data along with the local environments.

Finally, to identify key taxa driving the spatial and temporal variations in aquatic assemblages, we applied a similarity percentage analysis (SIMPER) to examine the contribution of each taxon to the differences in the whole aquatic assemblage of each season. We also carried out SIMPER to examine the seasonal change in aquatic assemblages and tested the significance of the seasonal decline or increase of each taxon from spring to autumn.

## 3 Results

**FIGURE 4:**
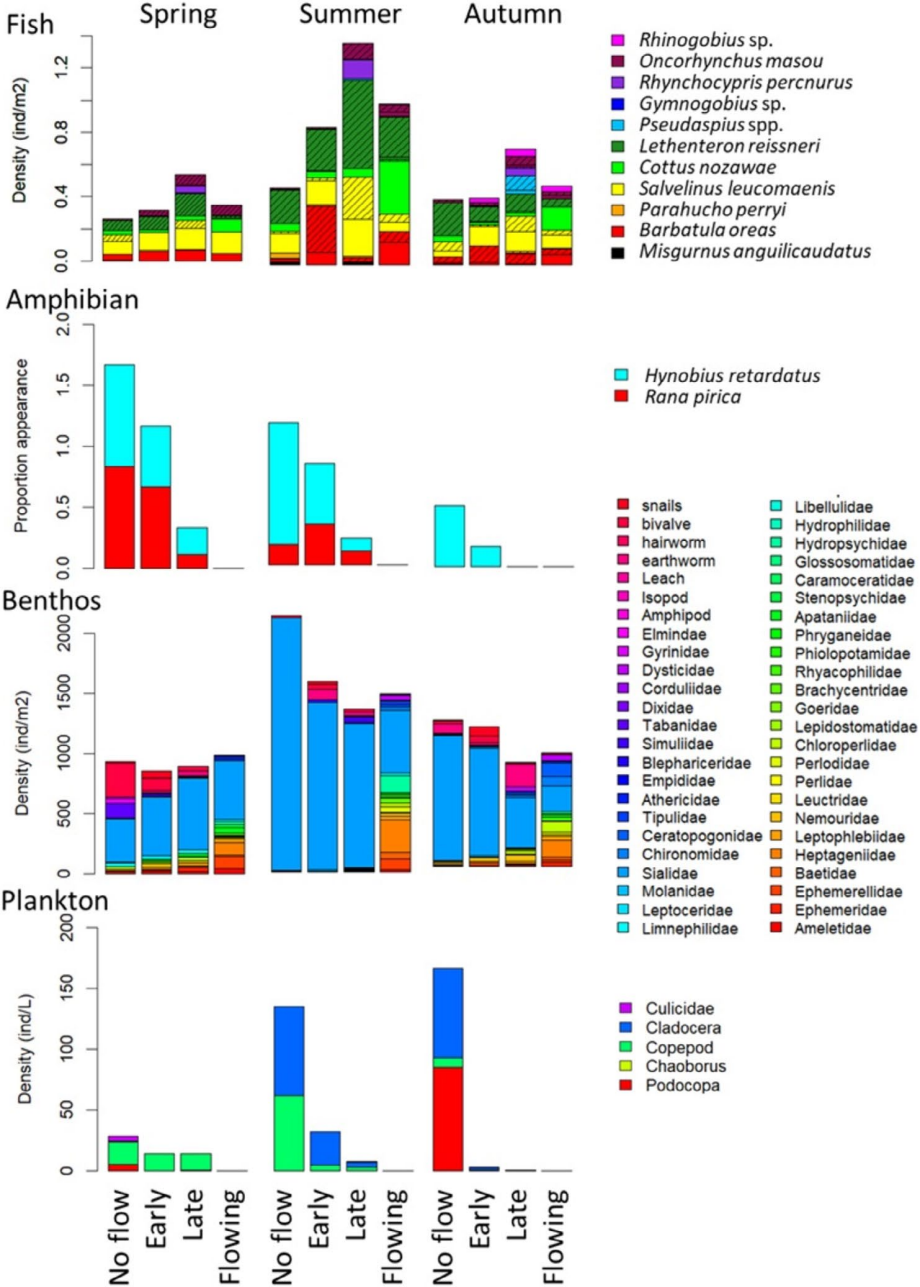
Spatial and seasonal variation of density and diversity of the four faunal groups. Each bar indicates the average density of each faunal group at multiple sites with the same connectivity to the river. The shaded portion of the fish figure indicates young of the year fish fry.

**FIGURE 5:**
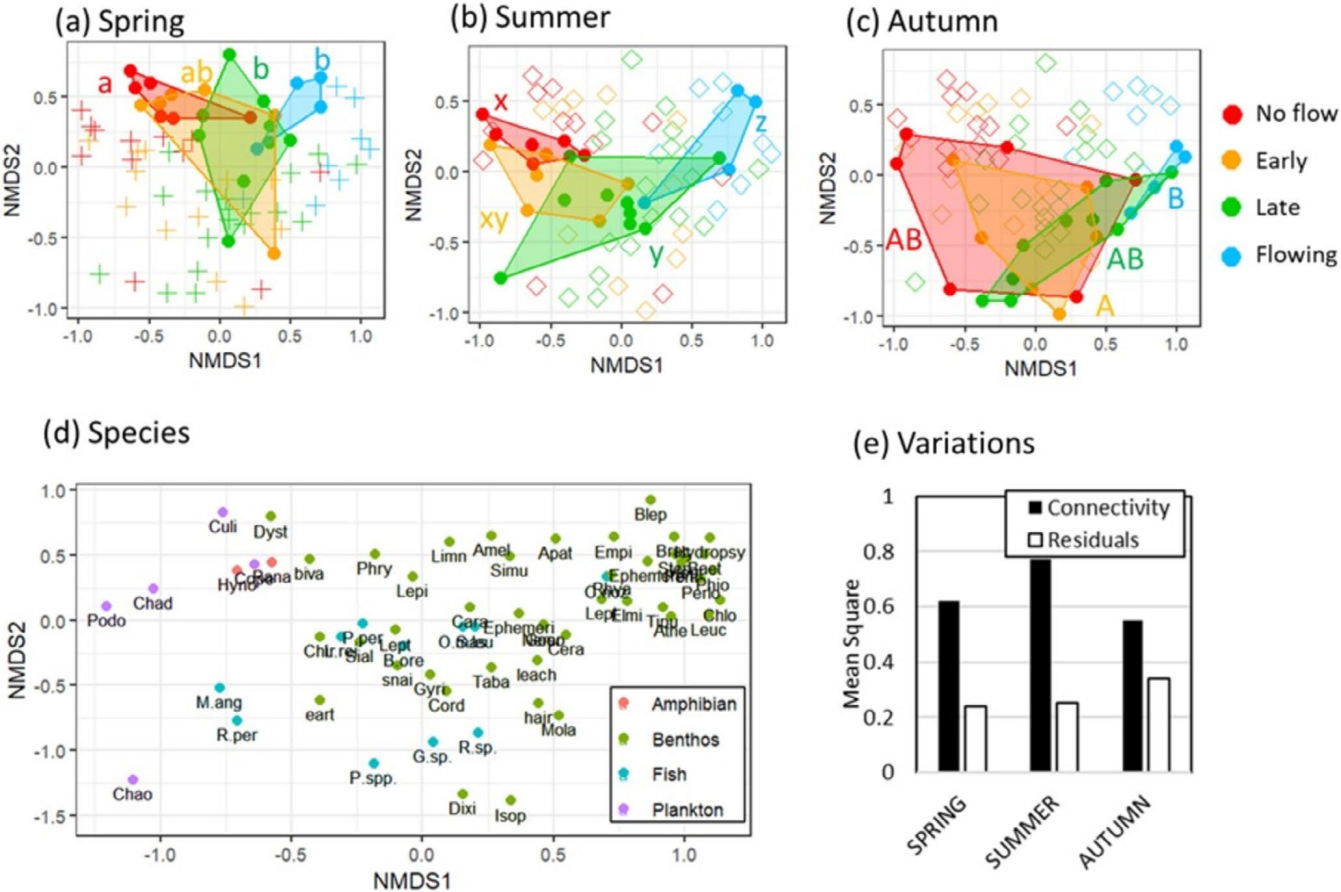
Non‐metric multidimensional scaling (NMDS) ordination of the whole aquatic biota (including the four faunal groups) at 25 sites in three seasons. Data for the four faunal groups were combined after standardization. Seventy‐five assemblage data were combined for the analysis, shown as points in (a–c) data from each season were coloured according to the connectivity category. Each polygon represents a convex hull created by connecting the outermost site scores for each of the four connectivity categories in each season. Different alphabet letters next to the polygons indicate significant differences among groups (*p* < 0.05; pairwise multilevel comparisons, *p*‐values were corrected using the Bonferroni method). (d) Species plots of the NMDS. Plots are coloured based on their faunal groups. Full taxonomic names of the abbreviations are in Table S4. (e) Mean square of variations between connectivity categories and residuals in each season.

Overall, two amphibian species, 11 fish species, three plankton orders and 48 benthic macroinvertebrate families were recorded in the three surveys. Assemblages of amphibians, fishes, macroinvertebrates and plankton varied with connectivity gradient and season (Figure 4, Tables S1–S4; two‐way PERMANOVA, *p* < 0.05). Consequently, the overall aquatic assemblage including all faunal groups was significantly influenced both by connectivity gradient and season, and their interaction was marginally significant (Figure 5, Table S2). In spring, ‘No‐flow’ pond assemblages were significantly different from ‘Late’ ponds and ‘Flowing’ sites. In summer, significant differences between ‘No‐flow’ pond assemblages and ‘Late’ ponds remained, and ‘Flowing’ site assemblages were significantly different from all other pond sites. In autumn, differences between ‘No‐flow’ pond and ‘Late’ pond assemblages became insignificant, and only the difference between ‘Early’ pond and ‘Flowing’ site assemblages was significant (Figure 5). In autumn, the mean square of variation was lower between categories and their residuals were higher.

**FIGURE 6:**
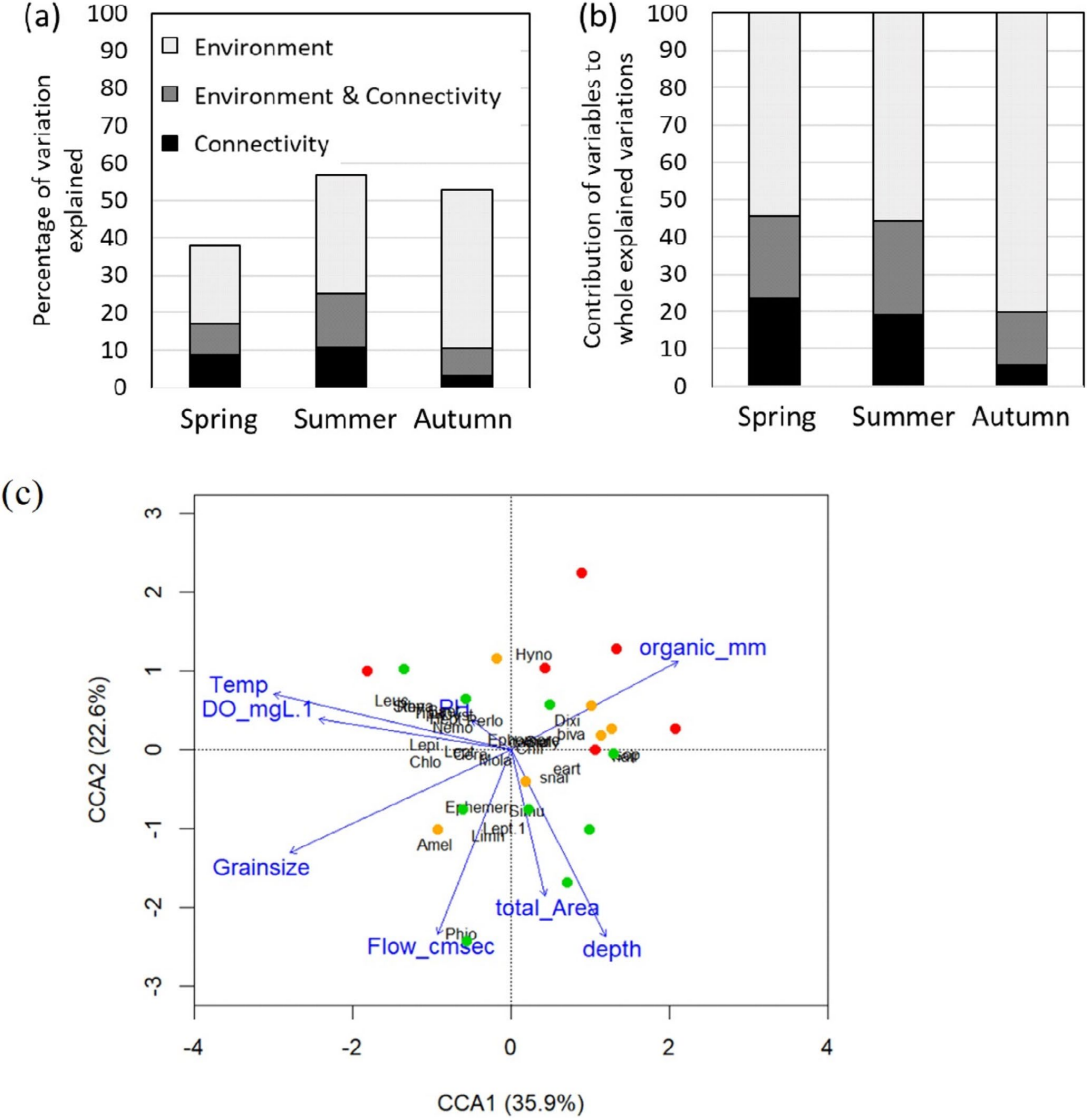
(a) Percentage of variation (β‐diversity: Bray–Curtis dissimilarity) explained by local environment and connectivity to the river in three seasons. (b) Proportion of the variation explained by each variable over all explained variations. (c) CCA plot, showing the correspondence (*~*influence) of local environmental factors (blue arrows; cf. Table 1) with the benthic macroinvertebrate and amphibian assemblages (dots) in autumn. The colours of dots represent connectivity categories of respective sites, showing no significant effect of the connectivity category on the assemblages. Four black letters show species plots. Full taxonomic names of the abbreviations are in Table S4. The analysis was significant overall at *F*_9,12_ = 1.56, *p* = 0.038 (permutation test for CCA).

Variation partitioning with generalised dissimilarity modelling showed that the factors driving the β‐diversity between sites change seasonally. The explained deviance of the aquatic assemblage was 38% in spring, 57% in summer and 53% in autumn (Figure 6a). Within the explained variation, the proportion explained by connectivity (pure connectivity or combination with environment) was 46% in spring, 44% in summer and then declined to 20% in autumn (Figure 6b). The importance of each environmental variable changed seasonally (Table S3). In spring, connection at the downstream end and area of ponds significantly influenced the assemblage, explaining 23% and 22% of the total deviance, respectively. In summer, dissolved oxygen, water temperature, area of ponds and connectivity at the downstream end significantly influenced the assemblage, explaining 11%, 9%, 8% and 3% of the total deviance, respectively. In autumn, area of ponds, flow velocity, DO, temperature, and grain size significantly influenced the assemblage, explaining 28%, 13%, 7%, 6% and 3% of the total deviance, respectively. Supplementary, we conducted variation partitioning with generalised dissimilarity modelling for benthic macroinvertebrates, fishes and plankton in the same manner (we could not apply the analysis to amphibians because of data limitations) and confirmed that the contribution of connectivity to the whole variation declined across seasons for all faunal groups (Figure S1). CCA analysis of the amphibious organisms (benthic macroinvertebrates and amphibians) in autumn visually explained the effects of local environmental factors. CCA1 explained 35.9% of the variation and was largely associated with properties of the water, including water temperature and dissolved oxygen level. CCA2 explained 22.6% of the variation and was largely associated with physical properties of the waterbodies such as total area, depth and flow. The effects of hydrological connectivity were not observed in the CCA plot. The permutation test showed a significant influence of local environmental factors on the assemblages (*F*_9,12_ = 1.56, *p* = 0.038) (Figure 6c).

SIMPER analysis showed seasonal changes in the taxa contributing to the assemblage difference along hydrological gradients (Table 2). Notably, the simper analysis shows that the three taxa, *Hynobius retardatus* , *Rana pirica* and Limnephilidae, which primarily contributed to the variation between ‘No flow’ and ‘Early’ as well as the variation between ‘Early’ and ‘Late,’ decreased by summer and then autumn, potentially causing loss in variation between those habitats in autumn. Furthermore, Leptophlebiidae and Nemouridae, which were the two primarily important taxa creating the differences between all ponds and flowing sites in summer, were both present in ponds and flowing sites in spring but disappeared only in ponds by summer. This led to the clear difference between the ponds and flowing site assemblages.

**TABLE 2:** Simper analysis showing the top 10 contributing taxa to the difference between connectivity categories for each season. Full taxonomic names of the abbreviations are given in Table S4.

Spring					Summer						Autumn						
Name	Group	Trend	Average	Rate	*p*	Name	Group	Trend	Average	Rate	*p*	Name	Group	Trend	Average	Rate	*p*
‘No flow’ to ‘Early’																	
Hyno	Amph	Mid	0.04	0.60	0.32	Hyno	Amph	Mid	0.07	0.50	0.17	Hyno	Amph	Mid	0.06	0.33	0.02*
Limn	Ben	Down	0.04	1.06	0.07	Rana	Amph	Down	0.05	2.00	0.11	*L. rei*	Fish	Mid	0.04	0.44	0.04*
Rana	Amph	Down	0.04	0.80	0.82	Chir	Ben	Mid	0.05	0.66	0.02*	Clad	Pla	Mid	0.04	0.02	0.02*
Cord	Ben	Mid	0.03	0.00	0.29	Sial	Ben	Mid	0.05	1.83	0.06	Chir	Ben	Mid	0.04	0.95	0.14
*B. ore*	Fish	Mid	0.03	1.57	0.28	Snai	Ben	Mid	0.04	inf	0.03*	Sial	Ben	Mid	0.04	Inf	0.14
Chir	Ben	Mid	0.03	1.33	0.24	Biva	Ben	Mid	0.04	3.83	0.16	Taba	Ben	Mid	0.03	1.88	0.32
Sial	Ben	Mid	0.03	0.50	0.04*	Clad	Pla	Mid	0.04	0.37	0.02*	Cord	Ben	Mid	0.03	2.50	0.08
Biva	Ben	Mid	0.03	0.36	0.02*	Culi	Pla	Mid	0.04	1.14	0.42	Snai	Ben	Mid	0.03	14.17	0.02*
*S. leu*	Fish	Mid	0.02	0.87	0.72	Cope	Pla	Mid	0.03	0.07	0.01**	Biva	Ben	Mid	0.03	3.05	0.04*
Podo	Pla	Mid	0.02	0.02	0.01**	*S. leu*	Fish	Mid	0.03	1.04	0.73	*B. ore*	Fish	Mid	0.03	2.61	0.34
‘Early’ to ‘Late’																	
Rana	Amph	Down	0.06	0.17	0.02*	Hyno	Amph	Mid	0.07	0.22	0.17	Sial	Ben	Mid	0.04	0.38	0.03*
Hyno	Amph	Mid	0.05	0.44	0.16	Rana	Amph	Down	0.05	0.33	0.14	Eart	Ben	Mid	0.04	9.87	0.14
Limn	Ben	Down	0.04	1.02	0.03*	*S. leu*	Fish	Mid	0.05	2.23	0.02*	*P. per*	Fish	Mid	0.04	8.51	0.07
*B. ore*	Fish	Mid	0.03	1.09	0.06	Culi	Pla	Mid	0.04	1.08	0.21	Taba	Ben	Mid	0.04	0.58	0.11
Chir	Ben	Mid	0.03	1.22	0.18	Sial	Ben	Mid	0.04	0.30	0.10	Chir	Ben	Mid	0.04	0.50	0.13
*O. mas*	Fish	Mid	0.03	1.82	0.22	Biva	Ben	Mid	0.04	0.55	0.09	*O. mas*	Fish	Mid	0.04	3.05	0.22
*S. leu*	Fish	Mid	0.03	1.69	0.57	Snai	Ben	Mid	0.04	0.07	0.02*	*B. ore*	Fish	Mid	0.03	0.58	0.09
Lept	Ben	Mid	0.02	1.47	0.21	*O. mas*	Fish	Mid	0.04	5.93	0.06	Snai	Ben	Mid	0.03	0.09	0.00**
Cord	Ben	Mid	0.02	inf	0.54	Eart	Ben	Mid	0.03	0.34	0.02*	*S. leu*	Fish	Mid	0.03	1.69	0.11
*P. per*	Fish	Mid	0.02	0.54	0.05	*P. spp*.	Fish	Mid	0.02	Inf	0.23	Limn	Ben	Down	0.03	2.00	0.16
‘Late’ to ‘Flowing’																	
Ephemere	Ben	Mid	0.04	24.75	0.00**	Lept	Ben	Mid	0.06	5.75	0.01**	Ephemeri	Ben	Down	0.06	5.63	0.02*
Hept	Ben	Mid	0.03	36.00	0.00**	Nemo	Ben	Mid	0.04	3.82	0.09	Hydropsy	Ben	Mid	0.04	Inf	0.00**
Empi	Ben	Down	0.03	18.00	0.01**	Cera	Ben	Up	0.04	45.00	0.00**	Rhya	Ben	Up	0.04	6.47	0.00**
Perlo	Ben	Mid	0.03	6.75	0.01*	Hept	Ben	Mid	0.04	90.90	0.00**	Sten	Ben	Mid	0.04	5.06	0.04*
Amel	Ben	Down	0.03	2.57	0.16	Chlo	Ben	Mid	0.04	56.25	0.00**	Tipu	Ben	Up	0.04	8.80	0.00**
*S. leu*	Fish	Mid	0.03	0.76	0.54	Rhya	Ben	Up	0.03	Inf	0.00**	Ephemere	Ben	Mid	0.04	1.97	0.17
*C. noz*	Fish	Mid	0.03	3.04	0.34	Phil	Ben	Mid	0.03	Inf	0.00**	Cera	Ben	Up	0.03	6.50	0.05
Apat	Ben	Down	0.03	2.25	0.04*	Elmi	Ben	Mid	0.03	5.71	0.02*	Lept	Ben	Mid	0.03	1.66	0.21
Brac	Ben	Mid	0.02	3.38	0.04*	Athe	Ben	Mid	0.03	4.31	0.02*	Eart	Ben	Mid	0.03	0.08	0.56
Sten	Ben	Mid	0.02	20.25	0.01**	*S. leu*	Fish	Mid	0.03	0.29	0.84	*P. per*	Ben	Mid	0.03	0.37	0.41

Abbreviations: Amel, ameletidae; Amph, amphibian; amphi, amphipod; Apat, apataniidae; Athe, athericidae; B.ore, *Barbatula oreas*; Baet, baetidae; Ben, benthos; biva, bivalve; Blep, blephariceridae; Brac, brachycentridae; C.noz, *Cottus nozawae*; Cara, caramoceratidae; Cera, ceratopogonidae; Chao, chaoborus; Chir, chironomidae; Chlo, chloroperlidae; Clad, cladocera; Cope, copepod; Cord, corduliidae; Culi, culicidae; Dixi, dixidae; Dyst, dysticidae; eart, earthworm; Elmi, elmindae; Empi, empididae; Ephemere, ephemerellidae; Ephemeri, ephemeridae; G.sp, *Gymnogobius* sp.; Glos, glossosomatidae; Goer, goeridae; Gyri, gyrinidae; hair, hairworm; Hept, heptageniidae; Hydrophi, hydrophilidae; Hydropsy, hydropsychidae; Hyno, *Hynobius retardatus*; Isop, isopod; L.rei, *Lethenteron reissneri*; leach, leach; Lepi, lepidostomatidae; Lept, leptoceridae; Lept, leptophlebiidae; Leuc, leuctridae; Libe, libellulidae; Limn, limnephilidae; M.ang, *Misgurnus anguillicaudatus*; Mola, molanidae; Nemo, nemouridae; O.mas, *Oncorhynchus masou*; P.per, *Parahucho perryi*; P.spp, *Pseudaspius* spp.; Perli, perlidae; Perlo, perlodidae; Phil, philopotamidae; Phry, phryganeidae; Pla, plankton; Podo, podocopa; R.per, *Rhynchocypris percnurus sachalinensis*; R.sp, *Rhinogobius* sp; Rana, *Rana pirica*; Rhya, Rhyacophilidae; S.leu, *Salvelinus leucomaenis*; Sial, sialidae; Simu, simuliidae; snai, snails; Sten, stenopsychidae; Taba, tabanidae; Tipu, tipulidae. ”*” and “**” indicate *p* < 0.05 and *p* < 0.01 respectively.

## 4 Discussion

The results of this study clearly demonstrate a seasonal shift in floodplain aquatic community structure across seasons. Some previous studies emphasise the hydrological connectivity as a primary driver of the floodplain community (Tockner, Malard, and Ward 2000; Gallardo et al. 2014) and others identify local environments as drivers of the variations in floodplain community between waterbodies (López‐Delgado, Winemiller, and Villa‐Navarro 2020). This study shows that both hydrological connectivity as a landscape factor and local environments drive the spatial structures of the floodplain community at different times of the year. The post‐flood aquatic communities were largely shaped by the landscape hydrological connectivity gradient as ecological legacies of the hydrological dynamics during the flood recession, as described in Uno et al. (2022). Variabilities of the aquatic communities between floodplain waterbodies remained across time, but the variability associated with the connectivity decreased over time and variabilities associated with local environments, including the properties of the water (cf. water temperature, dissolved oxygen level) and the physical properties of the waterbody (cf. depth and area). The shift in spatial structures of the floodplain aquatic communities occurred as a result of behavioural and life history movements of animals, common traits among animals adapted to the dynamic floodplain environment (Whiles and Goldowitz 2001; Jeffres, Opperman, and Moyle 2008; Holgerson et al. 2019; Acosta et al. 2020). Overall, the present study demonstrates how dynamically community structures can shift seasonally in such a seasonal ecotone as floodplain.

Amphibious animals, which have both aquatic and terrestrial lives, were major members of the studied floodplain aquatic community, including two amphibian species and 41 aquatic insect families. Taxa‐level analysis showed large contributions of them to the seasonal shift of the floodplain aquatic community structure. In spring, *Rana pirica* (frog) and *Hynobius retardatus* (salamander), and Limnephilidae (caddisfly) and diverse aquatic insect taxa contributed to the β diversity along the hydrological connectivity gradient. We have previously shown that their habitat selection during the flood recession period aligns their spatial distribution along a hydrological connectivity gradient after the flood (Uno et al. 2022). In summer, as oxygen at many lentic habitats drops to the threatening level for many benthic aquatic animals (Gaufin, Clubb, and Newell 1974), many amphibians and aquatic insect taxa disappeared from the ponds. For many amphibian and aquatic insect taxa, their disappearance from the water indicates their emergence to land as adults as adaptation to the seasonal character of the floodplain waterbodies (Kishida et al. 2014; Kawai and Tanida 2005). In contrast, some taxa, such as Apataniidae, Nemouridae, Leptophlebidae and Ameletiidae, disappeared only in ponds but not in the flowing sites, implying their selective mortality in ponds due to low oxygen level (if not other factors such as predation). For animals that primarily inhabit the lotic environment, floodplain aquatic waterbodies whose flow ceases in time may function as ecological traps. Some *Hynobius retardatus* were left in the waterbody in summer and autumn after most individuals left the water, which a previous study indicates are overwinter populations exhibiting phenotypic plasticity response to species interactions (Kishida et al. 2014). In autumn, other aquatic insects such as Ephemeridae, Ephemerellidae, Leptophlebiidae and Nemouridae appeared in the waterbodies. Most of these taxa as terrestrial winged adults oviposit their eggs in water during the summer low flow period. Therefore, the oviposition choice by the winged adults, based on the local environment during the summer low flow and environmental filtering for the remaining assemblages, has likely shifted the aquatic assemblages in floodplain ponds based on a stronger association with local environmental conditions than with the connectivity gradient.

Seasonal shifts of other members, plankton and fishes, were not detected in the taxa‐level analysis. Plankton (three taxonomic groups) was instead quantitatively responding to the local environmental change, and the effects of hydrological connectivity were not detected in this study. Their short life cycles probably explain that their response to the environmental shift occurs as a result of their reproduction in each habitat, rather than spatial movements. On the other hand, many fishes were ontogenetically shifting their habitat based on the body size of individuals occupying different habitats, as described well elsewhere (Bellmore et al. 2013; Jeffres, Opperman, and Moyle 2008). Among 11 species observed in our study, *Rhynchocypris percnurus* and *Misgurnus anguilicaudata* seemed to complete their life history in floodplain ponds as individuals with all life stages, including juvenile to mature adults, found in floodplain ponds. In contrast, *Cottus nozawae* was only in sites with constant flow. Eight other taxa, including *Rhinogobius* sp., *Oncorhynchus masou* , *Gymnogobius* sp., *Pseudaspius* spp., *Lethenteron reissneri* , *Salvelinus leucomaenis* , *Parahucho perryi* and *Barbatula oreas*, temporarily occupied the floodplain ponds as juveniles. In floodplain ponds, we observed their juveniles, including young of the year individuals and older cohorts, but not mature adults. Many of their mature adults were observed in the mainstem river and/or downstream lake. Our observations indicate these fish species are using the floodplain waterbodies as nurseries for the juvenile fishes (Endo et al. 2023). Because juveniles of these fishes occupy the floodplain waterbodies for more than 1 year and some cohorts of respective species stayed in the waterbodies all year, their movements did not contribute to the taxa‐level reassembly across seasons. Seasonal movements of fishes between floodplain waterbodies and rivers need to be determined with more careful observation of their cohorts or direct observations of their movements (Kanno et al. 2022).

The seasonal shifts in the spatial community structures described in this study should be widespread important mechanisms to support diverse biota in spatially and temporally dynamic seasonal ecotones represented by natural floodplains. Although many floodplain rivers have lost their natural flow dynamics and spatial heterogeneity, this study highlights their roles in the maintenance of the biodiversity. We identified the following environmental characters as key components of these processes: (1) seasonal flow regime and two spatial structures, (2) co‐existence of floodplain waterbodies with different hydrological connectivities to the river mainstem during floods and (3) waterbodies with diverse local environments in terms of size and water properties (temperature and dissolved oxygen). The variation in the water temperature and dissolved oxygen in natural floodplains may be associated with groundwater inputs (Ward et al. 1999). Hence, there is a possibility that what is considered the local environment in this study is also partly associated with hydrological connectivity by groundwater. While the increasing number of conservation and/or restoration of floodplain rivers take into account hydrology (King, Tonkin, and Mahoney 2009; Pander et al. 2019; Quinn, Hillman, and Cook 2000) and/or geomorphological structures (Powers, Helstab, and Niezgoda 2019) of floodplains, it is rare that they are considered in combination. This study emphasises the importance that the three environmental characters listed above are targeted altogether in floodplain river managements.

Our study highlighted seasonal dynamics of spatial community structures. Although community structures of a given ecosystem are commonly measured and diagnosed by one‐time spatial surveys, this study shows that the spatial structure of communities may change temporally, particularly in such dynamic systems as ecotones. Spatial heterogeneities of the environment at different times of the year can interact, and the spatial connectivity can also restrict the transition of the seasonal habitat shift of animals across seasons. The temporal and spatial dynamics of the natural environment structure the communities in combination, and their holistic understanding would be required for the understanding of such dynamic ecosystems. Furthermore, while this study only examined changes in the spatial patterns, the communities in one season would interact with those in another season as ecological legacies, through various factors such as population dynamics of each member of the community, species interactions and nutrient dynamics (Richardson 1991; Power, Parker, and Dietrich 2008; Yang 2008). Further studies would show spatially and temporally interacting natural community structures in dynamic environments.

## Author Contributions

**Hiromi Uno:** conceptualization (lead), data curation (lead), formal analysis (lead), funding acquisition (lead), investigation (lead), methodology (lead), project administration (lead), resources (lead), software (lead), supervision (lead), validation (lead), visualization (lead), writing – original draft (lead), writing – review and editing (lead). **Shunsuke Utsumi:** conceptualization (supporting), formal analysis (supporting), investigation (supporting), writing – review and editing (supporting). **Kentaro Morita:** conceptualization (supporting), investigation (supporting), writing – review and editing (supporting). **Osamu Kishida:** conceptualization (supporting), investigation (supporting), project administration (supporting), writing – review and editing (supporting). **Md. Khorshed Alam:** conceptualization (supporting), investigation (supporting), writing – review and editing (supporting). **Junjiro Negishi:** conceptualization (supporting), investigation (supporting), project administration (supporting), supervision (supporting), writing – review and editing (supporting).

## Ethics Statement

Our work conforms to the guidelines for the proper conduct of animal experiments in Japan. Field survey of aquatic animals was approved by the fishery and forestry department of Hokkaido (ID 610).

## Consent

The authors have nothing to report.

## Conflicts of Interest

The authors declare no conflicts of interest.

## Supporting information


Figure S1.

Tables S1–S4.

## Data Availability

All data related to this paper are available in Dryad (DOI: 10.5061/dryad.gmsbcc2xh).
