# Supplementary figures and images for: Hydrological Connectivity and Local Environment Alternately Drive Spatial Structure of Floodplain Aquatic Community Across Seasons
Source: Ecol Evol. 2025 Feb 24;15(2):e70880. doi: 10.1002/ece3.70880 (PMC11850756; doi:10.1002/ece3.70880)

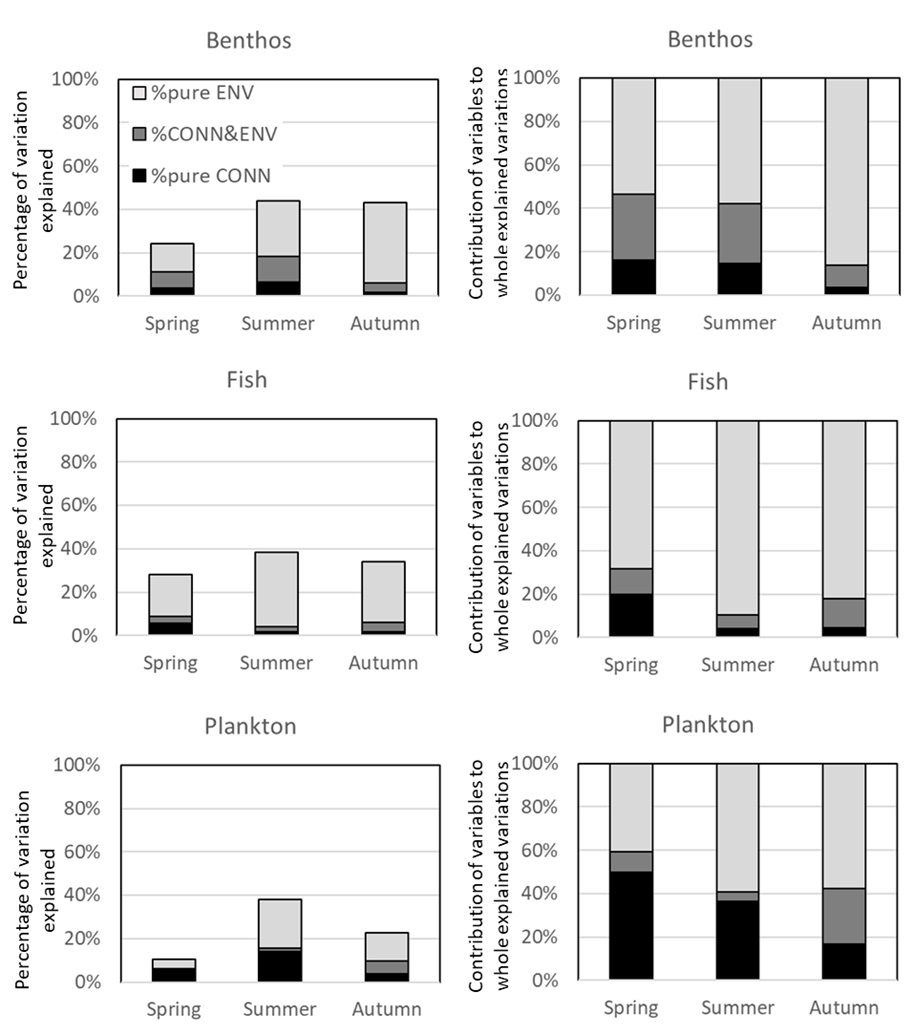

Supplement: Supplementary file 1 — Figure S1. [file ECE3-15-e70880-s002.jpg]
