# Supplementary material for: Hydrological Connectivity and Local Environment Alternately Drive Spatial Structure of Floodplain Aquatic Community Across Seasons
Source: Ecol Evol. 2025 Feb 24;15(2):e70880. doi: 10.1002/ece3.70880 (PMC11850756; doi:10.1002/ece3.70880)
Supplement: Supplementary file 2 — Tables S1–S4. [file ECE3-15-e70880-s001.zip › ece370880-sup-0002-TablesS1-S4/TableS1.docx]

Table S1:

| **Benthos** |  |  |  |  |  |  |  |  |
| --- | --- | --- | --- | --- | --- | --- | --- | --- |
|  | Df | SS | MeanSq | F.Model | R2 | Pr(>F) |  |  |
| Connectivity | 3 | 2.86 | 0.95 | 3.09 | 0.10 | 0.000 |  | *** |
| Season | 2 | 3.31 | 1.66 | 5.37 | 0.12 | 0.000 |  | *** |
| Connectivity:Season | 6 | 1.98 | 0.33 | 1.07 | 0.07 | 0.084 |  | . |
| Residuals | 63 | 19.43 | 0.31 |  | 0.70 |  |  |  |
| Total | 74 | 27.59 |  |  | 1.00 |  |  |  |
|  |  |  |  |  |  |  |  |  |
| *Pairwise adonis* | Df | SS | MeanSq | F.Model | R2 | p.value | p.adjusted | sig |
| Early - Late | 1 | 0.38 |  | 1.09 | 0.02 | 0.355 | 1.000 |  |
| Early - Flowing | 1 | 1.77 |  | 5.54 | 0.17 | 0.001 | 0.006 | * |
| Early - No flow | 1 | 0.44 |  | 1.23 | 0.03 | 0.217 | 1.000 |  |
| Late - Flowing | 1 | 1.44 |  | 4.22 | 0.10 | 0.001 | 0.006 | * |
| Late - No flow | 1 | 0.49 |  | 1.33 | 0.03 | 0.157 | 0.942 |  |
| Flowing - No flow | 1 | 1.68 |  | 4.94 | 0.15 | 0.001 | 0.006 | * |
|  |  |  |  |  |  |  |  |  |
| *Pairwise adonis* | Df | SS | MeanSq | F.Model | R2 | p.value | p.adjusted | sig |
| Spring - Summer | 1 | 2.32 |  | 7.26 | 0.13 | 0.001 | 0.003 | * |
| Spring - Autumn | 1 | 1.82 |  | 5.30 | 0.10 | 0.001 | 0.003 | * |
| Summer - Autumn | 1 | 0.83 |  | 2.39 | 0.05 | 0.008 | 0.024 | . |
|  |  |  |  |  |  |  |  |  |
| **Plankton** |  |  |  |  |  |  |  |  |
|  | Df | SS | MeanSq | F.Model | R2 | Pr(>F) |  |  |
| Connectivity | 3 | 3.20 | 1.07 | 6.77 | 0.21 | 0.007 |  | ** |
| Season | 2 | 1.34 | 0.67 | 4.26 | 0.09 | 0.000 |  | *** |
| Connectivity:Season | 6 | 0.90 | 0.15 | 0.95 | 0.06 | 0.363 |  |  |
| Residuals | 63 | 9.91 | 0.16 |  | 0.65 |  |  |  |
| Total | 74 | 15.35 |  |  | 1.00 |  |  |  |
|  |  |  |  |  |  |  |  |  |
| *Pairwise adonis* | Df | SS | MeanSq | F.Model | R2 | p.value | p.adjusted | sig |
| Early - Late | 1 | 0.19 |  | 1.20 | 0.03 | 0.280 | 1.000 |  |
| Early - Flowing | 1 | 0.73 |  | 6.07 | 0.18 | 0.005 | 0.030 | . |
| Early - No flow | 1 | 0.89 |  | 3.55 | 0.09 | 0.015 | 0.090 |  |
| Late - Flowing | 1 | 0.31 |  | 3.19 | 0.08 | 0.019 | 0.114 |  |
| Late - No flow | 1 | 2.00 |  | 9.80 | 0.19 | 0.001 | 0.006 | * |
| Flowing - No flow | 1 | 2.49 |  | 13.38 | 0.32 | 0.001 | 0.006 | * |
|  |  |  |  |  |  |  |  |  |
| *Pairwise adonis* | Df | SS | MeanSq | F.Model | R2 | p.value | p.adjusted | sig |
| Spring - Summer | 1 | 0.77 |  | 3.48 | 0.07 | 0.018 | 0.054 |  |
| Spring - Autumn | 1 | 0.51 |  | 3.04 | 0.06 | 0.027 | 0.081 |  |
| Summer - Autumn | 1 | 0.73 |  | 3.76 | 0.07 | 0.015 | 0.045 | . |
|  |  |  |  |  |  |  |  |  |
| **Amphibian** |  |  |  |  |  |  |  |  |
|  | Df | SS | MeanSq | F.Model | R2 | Pr(>F) |  |  |
| Connectivity | 3 | 6.01 | 2.00 | 13.58 | 0.34 | 0.002 |  | ** |
| Season | 2 | 1.41 | 0.71 | 4.79 | 0.08 | 0.000 |  | *** |
| Connectivity:Season | 6 | 0.78 | 0.13 | 0.88 | 0.04 | 0.262 |  |  |
| Residuals | 63 | 9.30 | 0.15 |  | 0.53 |  |  |  |
| Total | 74 | 17.51 |  |  | 1.00 |  |  |  |
|  |  |  |  |  |  |  |  |  |
| *Pairwise adonis* | Df | SS | MeanSq | F.Model | R2 | p.value | p.adjusted | sig |
| Early - Late | 1 | 1.19 |  | 6.25 | 0.13 | 0.012 | 0.072 |  |
| Early - Flowing | 1 | 1.57 |  | 9.11 | 0.25 | 0.006 | 0.036 | . |
| Early - No flow | 1 | 0.93 |  | 3.88 | 0.10 | 0.037 | 0.222 |  |
| Late - Flowing | 1 | 0.15 |  | 1.64 | 0.04 | 0.278 | 1.000 |  |
| Late - No flow | 1 | 4.18 |  | 26.90 | 0.38 | 0.001 | 0.006 | * |
| Flowing - No flow | 1 | 4.11 |  | 34.75 | 0.55 | 0.001 | 0.006 | * |
|  |  |  |  |  |  |  |  |  |
| *Pairwise adonis* | Df | SS | MeanSq | F.Model | R2 | p.value | p.adjusted | sig |
| Spring - Summer | 1 | 0.00 |  | 0.01 | 0.00 | 0.948 | 1.000 |  |
| Spring - Autumn | 1 | 1.06 |  | 5.46 | 0.10 | 0.011 | 0.033 | . |
| Summer - Autumn | 1 | 1.05 |  | 5.07 | 0.10 | 0.026 | 0.078 |  |
|  |  |  |  |  |  |  |  |  |
| **Fish** |  |  |  |  |  |  |  |  |
|  | Df | SS | MeanSq | F.Model | R2 | Pr(>F) |  |  |
| Connectivity | 3 | 2.11 | 0.70 | 2.70 | 0.11 | 0.052 |  | . |
| Season | 2 | 0.84 | 0.42 | 1.61 | 0.04 | 0.000 |  | *** |
| Connectivity:Season | 6 | 0.59 | 0.10 | 0.38 | 0.03 | 0.969 |  |  |
| Residuals | 63 | 16.43 | 0.26 |  | 0.82 |  |  |  |
| Total | 74 | 19.97 |  |  | 1.00 |  |  |  |
|  |  |  |  |  |  |  |  |  |
| *Pairwise adonis* | Df | SS | MeanSq | F.Model | R2 | p.value | p.adjusted | sig |
| Early - Late | 1 | 0.53 |  | 2.06 | 0.05 | 0.023 | 0.138 |  |
| Early - Flowing | 1 | 0.61 |  | 2.62 | 0.09 | 0.013 | 0.078 |  |
| Early - No flow | 1 | 0.35 |  | 1.24 | 0.04 | 0.268 | 1.000 |  |
| Late - Flowing | 1 | 0.48 |  | 2.20 | 0.06 | 0.030 | 0.180 |  |
| Late - No flow | 1 | 1.08 |  | 4.07 | 0.09 | 0.001 | 0.006 | * |
| Flowing - No flow | 1 | 1.21 |  | 4.99 | 0.15 | 0.001 | 0.006 | * |
|  |  |  |  |  |  |  |  |  |
| *Pairwise adonis* | Df | SS | MeanSq | F.Model | R2 | p.value | p.adjusted | sig |
| Spring - Summer | 1 | 0.49 |  | 2.01 | 0.04 | 0.045 | 0.135 |  |
| Spring - Autumn | 1 | 0.40 |  | 1.55 | 0.03 | 0.123 | 0.369 |  |
| Summer - Autumn | 1 | 0.36 |  | 1.25 | 0.03 | 0.265 | 0.795 |  |
