# Supplementary material for: Hydrological Connectivity and Local Environment Alternately Drive Spatial Structure of Floodplain Aquatic Community Across Seasons
Source: Ecol Evol. 2025 Feb 24;15(2):e70880. doi: 10.1002/ece3.70880 (PMC11850756; doi:10.1002/ece3.70880)
Supplement: Supplementary file 2 — Tables S1–S4. [file ECE3-15-e70880-s001.zip › ece370880-sup-0002-TablesS1-S4/TableS2.docx]

Table S2:

| **All aquatic biota** |  |  |  |  |  |  |  |  |
| --- | --- | --- | --- | --- | --- | --- | --- | --- |
| **PERMANOVA table** |  |  |  |  |  |  |  |  |
|  |  |  |  |  |  |  |  |  |
|  | Df | SS | MeanSq | F.Model | R2 | Pr(>F) |  |  |
| Connectivity | 3 | 4.24 | 1.41 | 5.14 | 0.17 | 0.0001 |  | *** |
| Season | 2 | 2.37 | 1.18 | 4.31 | 0.09 | 0.0001 |  | *** |
| Connectivity:Season | 6 | 1.61 | 0.27 | 0.97 | 0.06 | 0.0503 |  | . |
| Residuals | 63 | 17.31 | 0.27 |  | 0.68 |  |  |  |
| Total | 74 | 25.52 |  |  | 1.00 |  |  |  |
|  |  |  |  |  |  |  |  |  |
|  |  |  |  |  |  |  |  |  |
| **SPRING** |  |  |  |  |  |  |  |  |
|  | Df | SS | MeanSq | F.Model | R2 | Pr(>F) |  |  |
| Connectivity | 3 | 1.87 | 0.62 | 2.63 | 0.27 | 0.0003 |  | *** |
| Residuals | 21 | 4.97 | 0.24 |  | 0.73 |  |  |  |
| Total | 24 | 6.84 |  |  | 1.00 |  |  |  |
|  |  |  |  |  |  |  |  |  |
| *Pairwise adonis* | Df | SS | MeanSq | F.Model | R2 | P-value | P adjusted | sig |
| Early - Late | 1 | 0.43 |  | 1.59 | 0.11 | 0.13 | 0.76 |  |
| Early - Flowing | 1 | 0.78 |  | 3.08 | 0.28 | 0.02 | 0.14 |  |
| Early - No flow | 1 | 0.15 |  | 0.67 | 0.06 | 0.71 | 1.00 |  |
| Late - Flowing | 1 | 0.54 |  | 2.15 | 0.16 | 0.02 | 0.10 |  |
| Late - No flow | 1 | 0.87 |  | 3.88 | 0.23 | 0.00 | 0.02 | . |
| Flowing - No flow | 1 | 1.05 |  | 5.82 | 0.42 | 0.01 | 0.03 | . |
|  |  |  |  |  |  |  |  |  |
|  |  |  |  |  |  |  |  |  |
| **SUMMER** |  |  |  |  |  |  |  |  |
|  | Df | SS | MeanSq | F.Model | R2 | Pr(>F) |  |  |
| Connectivity | 3 | 2.32 | 0.77 | 3.12 | 0.31 | 0.0001 |  | *** |
| Residuals | 21 | 5.21 | 0.25 |  | 0.69 |  |  |  |
| Total | 24 | 7.53 |  |  | 1.00 |  |  |  |
|  |  |  |  |  |  |  |  |  |
| *Pairwise adonis* | Df | SS | MeanSq | F.Model | R2 | P-value | P adjusted | sig |
| Early - Late | 1 | 0.44 |  | 1.53 | 0.11 | 0.12 | 0.74 |  |
| Early - Flowing | 1 | 0.93 |  | 3.47 | 0.30 | 0.00 | 0.02 | . |
| Early - No flow | 1 | 0.31 |  | 1.53 | 0.13 | 0.18 | 1.00 |  |
| Late - Flowing | 1 | 0.73 |  | 2.53 | 0.19 | 0.01 | 0.03 | . |
| Late - No flow | 1 | 1.06 |  | 4.49 | 0.26 | 0.00 | 0.01 | . |
| Flowing - No flow | 1 | 1.30 |  | 7.19 | 0.47 | 0.00 | 0.01 | . |
|  |  |  |  |  |  |  |  |  |
|  |  |  |  |  |  |  |  |  |
| **AUTUMN** |  |  |  |  |  |  |  |  |
|  | Df | SS | MeanSq | F.Model | R2 | Pr(>F) |  |  |
| Connectivity | 3 | 1.65 | 0.55 | 1.62 | 0.19 | 0.0058 |  | ** |
| Residuals | 21 | 7.13 | 0.34 |  | 0.81 |  |  |  |
| Total | 24 | 8.79 |  |  | 1.00 |  |  |  |
|  |  |  |  |  |  |  |  |  |
| *Pairwise adonis* | Df | SS | MeanSq | F.Model | R2 | P-value | P adjusted | sig |
| Early - Late | 1 | 0.34 |  | 0.96 | 0.07 | 0.53 | 1.00 |  |
| Early - Flowing | 1 | 0.77 |  | 2.55 | 0.24 | 0.00 | 0.02 | . |
| Early - No flow | 1 | 0.39 |  | 1.06 | 0.10 | 0.32 | 1.00 |  |
| Late - Flowing | 1 | 0.62 |  | 1.97 | 0.15 | 0.02 | 0.13 |  |
| Late - No flow | 1 | 0.56 |  | 1.54 | 0.11 | 0.08 | 0.46 |  |
| Flowing - No flow | 1 | 0.75 |  | 2.31 | 0.22 | 0.03 | 0.19 |  |
