# Supplementary material for: Hydrological Connectivity and Local Environment Alternately Drive Spatial Structure of Floodplain Aquatic Community Across Seasons
Source: Ecol Evol. 2025 Feb 24;15(2):e70880. doi: 10.1002/ece3.70880 (PMC11850756; doi:10.1002/ece3.70880)
Supplement: Supplementary file 2 — Tables S1–S4. [file ECE3-15-e70880-s001.zip › ece370880-sup-0002-TablesS1-S4/TableS3.docx]

Table S3:

| Spring |  |  |  |
| --- | --- | --- | --- |
|  | % deviance explained | P-value | permutation |
| total_Area | 21.9 | 0.00 | 50 |
| depth | 0.0 | 1.00 | 50 |
| Flow (cmsec^-1^) | 9.8 | 0.22 | 50 |
| downstream_connection | 22.7 | 0.00 | 50 |
| Grainsize | 2.2 | 0.30 | 50 |
| DO (mgL^-1^) | 7.1 | 0.14 | 50 |
| Temp | 5.3 | 0.28 | 50 |
|  |  |  |  |
| Summer |  |  |  |
|  | % deviance explained | P-value | permutation |
| total_Area | 8.3 | 0.04 | 50 |
| depth | 0.8 | 0.46 | 50 |
| Flow (cmsec^-1^) | 5.1 | 0.08 | 50 |
| downstream_connection | 2.6 | 0.02 | 50 |
| Grainsize | 0.4 | 0.34 | 50 |
| DO (mgL^-1^) | 10.7 | 0.02 | 50 |
| Temp | 9.3 | 0.02 | 50 |
|  |  |  |  |
| Autumn |  |  |  |
|  | % deviance explained | P-value | permutation |
| total_Area | 27.7 | 0.00 | 50 |
| depth | 1.1 | 0.42 | 50 |
| Flow (cmsec^-1^) | 12.6 | 0.00 | 50 |
| downstream_connection | 0.0 | 0.98 | 50 |
| Grainsize | 3.1 | 0.04 | 50 |
| DO (mgL^-1^) | 6.9 | 0.00 | 50 |
| Temp | 6.4 | 0.02 | 50 |
