# Supplementary material for: Hydrological Connectivity and Local Environment Alternately Drive Spatial Structure of Floodplain Aquatic Community Across Seasons
Source: Ecol Evol. 2025 Feb 24;15(2):e70880. doi: 10.1002/ece3.70880 (PMC11850756; doi:10.1002/ece3.70880)
Supplement: Supplementary file 2 — Tables S1–S4. [file ECE3-15-e70880-s001.zip › ece370880-sup-0002-TablesS1-S4/TableS4.docx]

Table S4:

| Abbreviation | Full name | Group |
| --- | --- | --- |
| Rana | *Rana pirica* | Amphibian |
| Hyno | *Hynobius retardatus* | Amphibian |
| M.ang | *Misgurnus anguillicaudatus* | Fish |
| B.ore | *Barbatula oreas* | Fish |
| P.per | *Parahucho perryi* | Fish |
| S.leu | *Salvelinus leucomaenis* | Fish |
| C.noz | *Cottus nozawae* | Fish |
| L.rei | *Lethenteron reissneri* | Fish |
| P.spp. | *Pseudaspius* spp. | Fish |
| R.per | *Rhynchocypris percnurus sachalinensis* | Fish |
| O.mas | *Oncorhynchus masou* | Fish |
| G.sp. | *Gymnogobius* sp. | Fish |
| R.sp. | *Rhinogobius* sp. | Fish |
| Podo | Podocopa | Plankton |
| Chao | Chaoborus | Plankton |
| Cope | Copepod | Plankton |
| Clad | Cladocera | Plankton |
| Culi | Culicidae | Plankton |
| Amel | Ameletidae | Benthos |
| Ephemeri | Ephemeridae | Benthos |
| Ephemere | Ephemerellidae | Benthos |
| Baet | Baetidae | Benthos |
| Hept | Heptageniidae | Benthos |
| Lept | Leptophlebiidae | Benthos |
| Nemo | Nemouridae | Benthos |
| Leuc | Leuctridae | Benthos |
| Perli | Perlidae | Benthos |
| Perlo | Perlodidae | Benthos |
| Chlo | Chloroperlidae | Benthos |
| Lepi | Lepidostomatidae | Benthos |
| Goer | Goeridae | Benthos |
| Brac | Brachycentridae | Benthos |
| Rhya | Rhyacophilidae | Benthos |
| Phil | Philopotamidae | Benthos |
| Phry | Phryganeidae | Benthos |
| Apat | Apataniidae | Benthos |
| Sten | Stenopsychidae | Benthos |
| Cara | Caramoceratidae | Benthos |
| Glos | Glossosomatidae | Benthos |
| Hydropsy | Hydropsychidae | Benthos |
| Hydrophi | Hydrophilidae | Benthos |
| Libe | Libellulidae | Benthos |
| Limn | Limnephilidae | Benthos |
| Lept | Leptoceridae | Benthos |
| Mola | Molanidae | Benthos |
| Sial | Sialidae | Benthos |
| Chir | Chironomidae | Benthos |
| Cera | Ceratopogonidae | Benthos |
| Tipu | Tipulidae | Benthos |
| Athe | Athericidae | Benthos |
| Empi | Empididae | Benthos |
| Blep | Blephariceridae | Benthos |
| Simu | Simuliidae | Benthos |
| Taba | Tabanidae | Benthos |
| Dixi | Dixidae | Benthos |
| Cord | Corduliidae | Benthos |
| Dyst | Dysticidae | Benthos |
| Gyri | Gyrinidae | Benthos |
| Elmi | Elmindae | Benthos |
| amphi | Amphipod | Benthos |
| Isop | Isopod | Benthos |
| leach | Leach | Benthos |
| eart | earthworm | Benthos |
| hair | hairworm | Benthos |
| biva | bivalve | Benthos |
| snai | snails | Benthos |
